# Evaluation of tuberculosis infection control measures implemented at primary health care facilities in Kwazulu-Natal province of South Africa

**DOI:** 10.1186/s12879-015-0773-7

**Published:** 2015-03-07

**Authors:** Ntambwe Malangu, Michah Mngomezulu

**Affiliations:** School of Public Health, Sefako Magatho University of Health Sciences, Medunsa Campus, Pretoria, South Africa

## Abstract

**Background:**

Tuberculosis (TB) is a global public health concern. It is even more so as its incidence seems to be increasing in South Africa. The aim of this study was to describe and compare the tuberculosis infection control measures implemented by facilities in Ugu and Uthungulu health districts of Kwazulu-Natal province.

**Methods:**

This was a cross-sectional survey based on a self-administered questionnaire and site visit observations. Data were collected from healthcare workers at 52 health facilities from the beginning of February to mid-March 2012. The facilities that completed the questionnaires were visited for site observations.

**Results:**

The mean age of participants was 44.7 ± 11.7 years of age, ranging from 22 to 66 years old; 89.1% of them were females and nurses. Overall, some 48.6% (18 out of 37) of aspects of tuberculosis infection control encompassing administrative, environmental, clinical and occupational health measures were complied with by at least 80% of facilities surveyed. The unfortunate outcome of this inadequate compliance was that 23 and 12 cases of nosocomial tuberculosis had been diagnosed among staff members respectively in Ugu and Uthungulu districts.

**Conclusions:**

Overall, it appears that at the facilities surveyed, less than 50% of tuberculosis infection control measures were complied with. This finding calls for appropriate interventions to be designed and implemented. These include the purchase and installation of environmental control systems; the implementation of administrative tuberculosis infection control measures at each facility together with the training of staff members on the strict adherence to preventive measures including the use of personal protective equipment.

## Background

Tuberculosis (TB) is a global public health concern. It is even more so as its incidence seems to be increasing in South Africa; from 2010 to 2013, the number of new cases increased from 981 to 1000 cases per 1000,000 people [1]. As a disease that primarily affects the lungs, it can be transmitted from person to person via the airborne particles during normal social interactions. Thus, it poses a serious risk to uninfected people who come into contact with infected but undiagnosed people. For this reason, health care workers are even at a greater risk as they meet such people on a routine basis as they perform their duties. Unfortunately, a number of studies from other countries have found that health care workers do not always have sufficient knowledge and the right attitude in order to adopt acceptable practices for preventing the spread of resistant tuberculosis and treating tuberculosis adequately [2-5].

Yet high rates of resistant tuberculosis have been notified for the Western Cape, Eastern Cape as well as KwaZulu-Natal (KZN) provinces. Moreover, there is evidence that resistant tuberculosis in South Africa is spread mostly by the transmission of MDR strains [6-10]. It is noted that, conceptually, several individual characteristics of healthcare workers, institutional and managerial systems in place as well as the regulatory frameworks affect the implementation of infection control measures [11,12].

Although several studies have been conducted about the prevalence and incidence of tuberculosis across several provinces of South Africa, no comparative studies have been conducted on tuberculosis infection control measures implemented at health facilities level particularly in Kwazulu-Natal province [13-15]. This study endeavored to fill this gap; its purpose was to describe and compare the tuberculosis infection control measures implemented by facilities in Ugu and Uthungulu health districts of Kwazulu-Natal province.

In doing so, this study sought to identify gaps that warrant further investigations and interventions. It is expected that the findings of this study may be used by health planners and institutional managers to review their tuberculosis infection control practices and design appropriate interventions to address the shortcomings identified.

## Methods

### Study design

This was a cross-sectional survey based on a self-administered questionnaire and site visit observations. This design was chosen because of its simplicity, affordability and ease of implementation [16].

### Population and data collection

Data were collected from healthcare workers at study sites from the beginning of February to mid-March 2012. In order to increase their participation, telephonic follow-up was conducted to encourage those who had not submitted their completed questionnaires to do so. The targeted study population of healthcare workers was from facilities as listed by the provincial department of Health for these two districts [17]. These two districts were chosen because of their rural location and for logistical reasons. Ugu Health District is found in the lower South Coast of the Province of KZN. At the time of the study, it had 5 hospitals and 38 fixed clinics. Similarly, Uthungulu Health District had 8 hospitals and 44 fixed Clinics. The hospitals were district hospital with beds capacities ranging from 160 to 500 beds; while clinics were all day facilities providing primary healthcare services.

A census of health facilities was conducted; the questionnaires were sent to institutional managers of all 95 facilities in the two health districts. The questionnaires were filled in by these institutional managers or the people they delegated to perform this task. The data collected from them were their socio-demographic details such as their age, sex and professional category; as well as their responses on questions about the tuberculosis infection control measures implemented at their facilities and on the number of healthcare workers at their facilities they knew had been diagnosed with TB during the last three years through occupational screening.

In addition, observational data were collected on the day of unannounced visits to the health facilities from which completed questionnaires had been received. Observations were made about building designs and air-conditioning systems, availability of personal protective equipment, documents such as tuberculosis infection control plans, TB registers, specimens tracing log books and several other documents. Data from observations were also used to corroborate some responses in the completed questionnaires. These observations were recorded on an observation form designed following the WHO guidelines on the prevention of tuberculosis in healthcare facilities [18]. Based on these guidelines, a framework of four indexes was developed as follows: ten questions or aspects each for the administrative, clinical and environmental control measures scores and 7 for the occupational health control measures scores. To comply fully with the WHO and the South African National Department of Health tuberculosis infection control guidelines, facilities needed to have implemented all (100%) infection control measures.

Data were captured into a spread sheet and imported into STATA (version 10) for data analysis. Data were captured by two people who checked each other by means of a printout. Though, traditionally, tuberculosis infection control measures have been classified into three sets of controls namely administrative, environmental and personal; for this study, the analysis was conducted using the framework described above in order to reflect the stage and locus of control by stakeholders. Administrative measures included all initiatives taken by institutional managers about the structures and systems in place to curtail tuberculosis transmission. These measures reflect the commitment of institutional managers with regard to decreasing the risks of tuberculosis transmission to themselves, their staff and their patients [19].

Hence these administrative measures include among others the establishment of relevant committees, the appointment of staff members to guide the infection control efforts, the provision and keeping of relevant registers, the development, implementation and review of tuberculosis control plans [20]. Closely aligned to the administrative measures are clinical control measures that aim to ensure that people with tuberculosis symptoms are promptly identified, separated and treated [21,22]. These controls included the availability of a cough symptom check, the education of patients on coughing etiquette as well as the separation of suspected tuberculosis patients. These clinical control measures reflect the performance of health care workers as they strive to protect themselves and other patients not yet infected with tuberculosis.

Environmental control measures, on the other hand, are about methods that are used to reduce the concentration of infectious agents in the air, and the methods to control the direction of potentially infectious air. They are related to the buildings’ design and construction. Some of these measures include adequate ventilation systems, airflow and air circulation directing systems as well as ergonometric postures taken by health care workers in relation to the airflow, and the existence of windows and ultraviolet irradiation equipment. The measures are strongly recommended for the prevention of the spread of tuberculosis within health facilities by the Department of Health of South Africa [23].

Finally, occupational health measures include the use of personal protective equipment such as N95 respiratory masks, the screening of staff members and the provision of prophylaxis treatment to immune-compromised staff members. The use of protective equipment is recommended for all health workers when caring for patients suspected of having infectious diseases such as tuberculosis [24,25].

### Data analysis

A simple scoring system was used: the assessment was made on the basis whether the systems, documents or committees were present or absent; or whether the specific control measures were implemented or not. From this scoring, the proportions of compliance were determined. A comparison was made based on the proportions scored by facilities in Ugu and Uthungulu districts; a statistical testing based on the independent samples *t*-test was performed with the level of statistical significance being set at p < 0.05 [26,27].

### Ethical considerations

This study was conducted after approval was obtained from the Medunsa Research Ethics Committee (MREC) of the University of Limpopo. Additionally, permission was requested and obtained from the provincial authorities in Kwazulu-Natal. Individual institutional managers consented to participate in the study; similarly, where individuals other than managers completed the questionnaires, they also consented to participate in the study.

## Results

A response rate of 54.7% was achieved as responses were received from 52 out of 95 health facilities. The mean age of participants was 44.7 ± 11.7 years of age, ranging from 22 to 66 years old. Although all healthcare workers indicated their age, some did not record their sex and professional category. Among the 46 participants who had indicated their sex, 41 (89.1%) of them were females. Similarly, all 43 healthcare workers who reported their professional category were nurses. Of the 52 healthcare workers, 50 (96.1%) worked at primary health clinics. Of the other two respondents from hospitals, one worked in Ugu; the other in Uthungulu districts respectively. Overall, the majority of healthcare workers, 27 (51.9%) of them were from Uthungulu district.

With regard to administrative control measures, the responses from healthcare workers and the findings during site visit observations are reported in Table 1.

**Table 1:** **Administrative control measures scores**

**Aspects Assessed**	**Ugu District (N = 25)**		**Uthungulu District (N = 27)**		**Overall**	
	**n**	**%**	**n**	**%**	**Total**	**%**
Existence of an infection prevention and control committee	17	70.8	22	81.5*	39	76.5
Evidence of committee members having met	17	68.0	19	70.4	36	69.2
Existence of a written infection control plan	12	48.0	23	85.2**	35	67.3
Evidence of training conducted in infection control in the last 6 months	16	69.6*	15	55.6	31	62.0
Existence of a TB infection control committee or sub-committee	13	54.2*	11	40.7	24	47.1
Nurses represented in the committee	14	56.0*	13	48.1	27	51.9
Existence of a designated person responsible for TB control	12	52.2	17	68.0*	29	59.6
Existence of a Written TB control plan	3	12.0	9	33.3*	12	23.1
Evidence that the TB control plan had been reviewed	5	20.0	7	25.9*	12	23.1
Existence of a register for TB suspects at the facility	25	100.0	26	96.3	51	98.1

** = higher and statistically significant * = high but not statistically significant.

Of the 10 administrative aspects assessed, all but one facility complied with the requirement of keeping a register for TB suspects. The compliance with the other 9 aspects ranged from 23.1% to 76.5%. The major shortcomings were the non-existence of a tuberculosis infection control committee and a tuberculosis control plan as well as the lack of evidence that the control plan had been reviewed. However, there were notable differences between the two districts: more facilities in Uthungulu district had a written infection control plan than in Ugu district (85.2% versus 48.0%, p = 0.001); and a written tuberculosis control plan (33.3% versus 12.0%, p = 0.07).

With regard to environmental control measures (Table 2), the three aspects that over 80% of facilities complied with were ensuring an unrestricted airflow in the working areas, the existence of ceilings that were at least 3 meters high and a designated area for sputum production by patients. Moreover, about half of the facilities had vents in addition to windows for air circulation. With regard to the remaining six aspects, most facilities did not comply: only two facilities had all windows that could be opened; three facilities had fans to increase air circulation in working areas, six had air directed in one direction, and nine facilities reported having their windows always being open during working hours. Sadly ultraviolet irradiation was used in high risk areas only in 20% of facilities. Staff members failed to comply with the requirement to sit with their backs towards the direction of airflow; only in 23.6% of facilities was this requirement complied with.

**Table 2:** **Environmental control measures scores**

**Aspects Assessed**	**Ugu District (N = 25)**		**Uthungulu District (N = 27)**		**Overall**	
	**n**	**%**	**n**	**%**	**Total**	**%**
Evidence that there is unrestricted airflow in wards	18	85.7	24	92.3	42	89.0
Evidence that the ceiling is high(3 meters or more)	19	95.0*	22	84.6	41	89.8
Existence of vents in addition to windows in TB wards	9	52.9	12	48.0	21	50.5
Evidence that all windows can be opened	1	4.0	1	3.7	2	3.9
Windows are always opened during working hours	2	8.0	7	25.9*	9	17.0
Existence of fans to increase air circulation in working areas	2	8.3*	1	3.7	3	6.0
Airflow is directed in one direction	2	8.0	4	16.7*	6	12.4
Staff members sit with their backs towards the direction of airflow	7	28.0*	5	19.2	12	23.6
Ultraviolet irradiation is used in high risk areas	5	22.7*	4	16.7	9	19.7
Existence of a designated area for sputum production by patients	23	92.0*	22	81.5	45	86.8

* = high but not statistically significant.

Although, only 17.0% of facilities had windows always kept open during working hours, more facilities in Uthungulu than in Ugu met this requirement (25.9% versus 8.0%, p = 0.09). Likewise, more facilities in Uthungulu than in Ugu district had air circulation directed in one direction (16.7% versus 8.0%, p = 0.31). Furthermore, even though only 5.9% of facilities used fans to increase air circulation; more facilities in Ugu than Uthungulu had fans (8.3% versus 3.7%, p = 0.46). Similarly, more facilities in Ugu than in Uthungulu used ultraviolet light (22.7% versus 16.7%, p = 0.44).

With regard to clinical control measures, all facilities had complied with requirements about educating patients about the symptoms of tuberculosis and the teaching of coughing etiquette as shown in Table 3. Furthermore, at least 80% of facilities complied with having a cough symptom checklist, having a specimen tracking system in place as well as printed educational materials for patients and displaying coughing etiquette posters. However, on the remaining four criteria, most facilities did not comply. The most notable difference between facilities in the two districts was that no single facility in Ugu district reported separating patients suspected of tuberculosis from other patients but one facility in Uthungulu district reported doing so.

**Table 3:** **Clinical control measures scores**

**Aspects Assessed**	**Ugu District (N = 25)**		**Uthungulu District (N = 27)**		**Overall**	
	**n**	**%**	**n**	**%**	**Total**	**%**
Existence of a cough symptoms checklist for patients’ screening	21	87.5	25	92.6	46	90.0
Implementation of screening of patients for cough in the facility	3	12.5	3	11.5	6	12.0
Education of all patients entering the facility about cough hygiene	2	8.3	2	7.7	4	8.0
Provision of masks or hygiene tissue papers to patients coughing	4	16.7	4	15.4	8	16.0
Separation of TB suspects or patients from other non-infected patients	0	0.0	1	3.8	1	1.9
Education of patients that are coughing about coughing etiquette	25	100.0	27	100.0	52	100.0
Existence of a specimen tracking system at the facility	24	96.0	25	92.6	49	94.3
Education of patients about TB symptoms and prevention	25	100.0	27	100.0	52	100.0
Provision of printed educational materials to patients	22	88.0	26	96.3*	48	92.1
Existence of posters displaying coughing etiquette	20	80.0	25	92.6*	45	86.3

* = high but not statistically significant.

With regard to occupational health measures (Table 4), all facilities complied with the requirement about providing isoniazid prevention therapy to HIV-infected staff members; in addition, over 80% of facilities complied with the following requirements: making N95 masks available to staff members, training staff members on tuberculosis control practices and screening staff members for tuberculosis. However, less than 70% of facilities complied with the requirement of keeping training records.

**Table 4:** **Occupational health control measures**

**Aspects Assessed**	**Ugu District (N = 25)**		**Uthungulu District (N = 27)**		**Overall**	
	**n**	**%**	**n**	**%**	**Total**	**%**
Staff members use personal protection during sputum induction	17	68.0	18	66.7	35	67.3
Masks (N95) are available for use by staff members in risk areas	23	92.0*	21	77.8	44	84.9
Staff members trained about TB infection control practices	20	80.0	23	85.2*	43	82.6
Records are kept about the training conducted	18	72.0*	15	55.6	33	63.8
Staff members are screened for TB	22	88.0	24	88.9	46	88.4
Isoniazid preventive therapy is available to HIV-infected staff members	25	100.0	27	100.0	52	100.0
Facilities reporting a staff member infected with TB during the last 3 years	10	40.0	8	29.6	24	52.2

* = high but not statistically significant.

The screening of staff members for tuberculosis yielded for following findings: 10 facilities in Ugu district reported 23 cases of tuberculosis identified among staff members while 8 facilities in Uthungulu district reported 12 cases. Hence more facilities in Ugu than in Uthungulu district reported as having their staff members infected with tuberculosis during the last three years before the survey but the difference was not statistically significant (40.0% versus 29.6%, p = 0.45). On the contrary, more facilities in Ugu kept well the training records than the facilities in Uthungulu district (72.0% versus 55.6%, p = 0.24).

Overall, one out 10 aspects of administrative control measures was complied with by at least 80% of facilities in Ugu and Uthungulu districts. This aspect was the keeping of a register for patients suspected of tuberculosis. Moreover, three out 10 aspects of environmental control measures were complied with by over 80% of facilities. These aspects were ensuring an unrestricted airflow in the working areas, having ceilings that are at least 3 meters high, and having designated areas for sputum production by patients. In addition, four out of 7 aspects relating to occupational health controls were complied with by 80% or more facilities in both districts. Likewise, six out 10 aspects of clinical controls were complied with by over 80% of health facilities in both districts.

Taken together, less than half (48.6%) of the 37 aspects of tuberculosis infection control measures assessed were complied with by at least 80% of facilities surveyed. What is noteworthy is that all facilities complied with the following requirements: providing isoniazid preventive therapy to HIV-infected staff members; educating patients about the prevention, symptoms of tuberculosis and coughing etiquette. In addition, all facilities in Ugu district complied with the requirement of keeping a register for patients suspected of having tuberculosis. However, with regard to environmental control measures, there was no single aspect on which all facilities (100%) in both districts complied with.

## Discussion

Overall, this study shows that less than 50% of requirements for tuberculosis infection control were complied with by the facilities surveyed. It is a matter of concern that 9 of the 10 administrative control measures which fall under the responsibilities of institutional managers were not complied with. This finding suggests that district and facility managers should be called upon to take corrective actions in order to address these shortcomings. The findings from this study concur with reports by several other investigators who noted failures in administrative controls [28,29].

Similarly, the findings from the site visits observations showed that there were clearly building design issues as most facilities did not have windows that could be opened or fitted mono-directional airflow systems in place. There were also shortages of relevant equipments such as fans to increase air circulation in working areas and ultraviolet irradiation equipment in high risk areas. Our findings confirm the reports by Naidoo and co-workers who found that there were shortcomings relating to air circulation in facilities surveyed from the same province [30]. It is still the responsibility of health care managers to ensure that the required environmental systems and equipments are purchased and used. It is still unclear whether the failure for them to procure the required systems and equipments resulted from budgetary constraints, ignorance or unwillingness to do so [31].

Given the high burden of tuberculosis in the country and in the province where this study was conducted, there is a need for institutional managers to be compelled to ensure that the required equipments and systems are put in place in order to bolster tuberculosis infection control. This legal or regulatory intervention is required in order to ensure that its non-compliance could be enforced [32,33]. However, the finding that only 17.3% of facilities had windows kept open during working hours needs to be interpreted with caution because the survey was conducted at the beginning of winter. This may have contributed to the fact that windows were closed to protect patients from the cold weather.

Moreover, it was found that only one facility reported separating patients suspected of tuberculosis from the others. This precautionary measure is very important because of the possibility of tuberculosis transmission as an infected person is coughing profusely in the presence of other patients [34]. It could be speculated that this shortcoming could have contributed to the emergence of transmission of multi-drug resistant tuberculosis that occurred in the whole province of KwaZulu-Natal [35-37].

The outcomes of non-compliance with tuberculosis infection control measures are clearly reflected by the presence of 35 cases of nosocomial tuberculosis among staff members in the facilities surveyed from the two districts. This finding is consistent with reports from other investigators that tuberculosis is one of the most common occupational health infections in South Africa [38,39]. This finding calls for renewed efforts to ensure that health care workers are trained and provided with the necessary personal protective equipment as well as the rolling out of isoniazid preventive treatment for staff members who are HIV-positive [40-43].

The above findings must be considered in light of the following limitations of the study. Firstly, there was a low participation of hospitals; 1 (20%) out of 5 hospitals in Ugu district and 1 (12.5%) out of 8 hospitals in Uthungulu district participated in this study. Secondly, the overall response rate of about 55% is low but acceptable because it is well established that the participation of healthcare workers in surveys is generally low. Moreover, the lack of incentives of any kind may have contributed to this low response rate [44,45]. Thirdly, upon telephonic enquiry on why some institutional managers did not complete the questionnaires, the most common excuse was that they were too busy to find time to complete the questionnaires. Fourthly, it seems that some institutional managers may have refused to participate simply because they may have wrongly perceived this study as an inspection of their performances or had fears about the use of its findings particularly because it was clearly stated that unannounced visits will be conducted subsequently [46]. Fifthly, as a cross-sectional study, the findings show a snapshot of the situation at the time of the survey, hence they do not reflect a permanent feature at the facilities surveyed. Finally, it is possible that social desirability may have led some healthcare workers to express views that do not accurately reflect the actual situation at their facilities [47,48].

The preceding arguments suggest that the results presented above reflect an incomplete picture of the situation regarding the implementation of tuberculosis infection control measures at health facilities in the two districts of KwaZulu-Natal Province. There is clearly a need for a comprehensive audit of all health facilities and more studies particularly targeting district hospitals and higher level of health care facilities such as regional and academic hospitals. Nonetheless, the findings from the study point out to the shortcomings that should be addressed to curtail nosocomial transmission of tuberculosis [49].

## Conclusions

In conclusion, overall, it appears that at the facilities surveyed, less than 50% of tuberculosis infection control measures were complied with. Consequently, 35 cases of tuberculosis had been reported among health care workers. Several interventions are required such as a complete audit at all health care facilities; the purchase and installation of environmental control systems; the implementation of administrative tuberculosis infection control measures at each facility together with the training of staff members on the strict adherence to preventive measures including the use of personal protective equipment.
